# Sources of Intelligibility of Distant Languages: An Empirical Study

**DOI:** 10.1177/00238309251345952

**Published:** 2025-07-11

**Authors:** Jiří Milička, Anna Marklová, Michal Láznička, Vojtěch Diatka, Hana Bednářová, Jiří Matela, Michal Škrabal

**Affiliations:** Department of Linguistics, Faculty of Arts, Charles University, Czech Republic; Department of Linguistics, Faculty of Arts, Charles University, Czech Republic; Independent Researcher, Prague, Czech Republic; Institute of Czech Language and Theory of Communication, Faculty of Arts, Charles University, Czech Republic; Department of Japanese Studies, Faculty of Arts, Masaryk University, Czech Republic; Department of Linguistics, Faculty of Arts, Charles University, Czech Republic

**Keywords:** Intelligibility, iconicity, systematicity, linguistic affinity

## Abstract

Research into iconicity, systematicity, and sound-symbolism has revealed that the connection between linguistic form and meaning is not completely arbitrary. In the present study, native Czech speakers, unfamiliar with Hindi, were presented with a task in which they had to match Hindi words with their corresponding Czech translations. The words were randomly selected from a Hindi corpus. Despite the considerable linguistic gap between the two languages, the analysis showed that the Czech participants were able to accurately discern the meanings of approximately 60% of the Hindi word pairs, surpassing the 50% success rate that would be expected by random guessing alone. This experiment was subsequently replicated using Turkish, Japanese, and Latvian words, demonstrating the robustness of this phenomenon across different languages. In the case of a closer language like Latvian, the success rate reached 80%. However, even a distant language such as Japanese reached the 60% success level. Furthermore, the study explored potential factors influencing intelligibility. Data collected from a total of 1,128 participants found that the phonological similarity of Czech words and their translation, word length alignment, presence of cognates, and the way the trials were presented had a significant effect on the success rate of guessing the correct translation across all four languages. In addition, language-specific effects were identified.

## 1 Introduction

Let us imagine a world where the assignment of meanings to words is entirely arbitrary, devoid of any inherent connection between the sequences of phonemes or letters and their associated meanings. Such a world may not be that difficult to imagine; for example, the word “dog” does not contain in its form any characteristics of dogs (de Saussure, 2011), and even onomatopoeic words vary across languages. In this hypothetical world, if we were given two words from an unfamiliar language, and someone provided us with their meanings but did not specify which meaning corresponds to which word, the probability of us correctly deducing the meanings would be no better than a coin toss, standing at 50%. However, such a world does not exist. Instead, languages frequently exhibit deviations from complete arbitrariness through various clues that assist in meaning deduction. This study investigates which of these clues are most pivotal in helping people infer the meanings of unknown words. To examine this, we use word pairs randomly selected from a corpus, allowing us to analyze how individuals interpret naturally occurring linguistic data rather than artificially constructed examples. By doing so, we aim to determine whether participants rely primarily on phonetic, morphological, or semantic similarities when guessing word meanings.

The fact that language users are able to associate certain meanings with certain forms more readily than others, particularly when presented with alternatives, has been typically discussed in the context of sound iconicity. When the form of a word depicts or reflects some facets of its meaning, it is easier to guess this meaning. Summarizing a vast body of literature on sound iconicity in language, Winter et al. (2024) take iconicity to be a subjective property both on the part of language production and perception, and they define iconicity as:

A signal in any medium or modality, such as a word, sign, or gesture, is iconic to the extent that language users produce or perceive it through a sense of resemblance between some aspect of its form and some aspect of its meaning.

According to Emmorey (2014), this “sense of resemblance” is best understood as a structural mapping, that is, a structural alignment between two representations (semantic and acoustic) resulting in the cognitive process of comparison (see also Taub, 2001; Wilcox, 2004).

Despite the fact that the idea that at least some forms of words may reflect their meanings goes back to at least Plato as well as the fact that first behavioral research into linguistic iconicity dates to the first half of the 20th century (Köhler, 1929; Sapir, 1929), systematic research into iconicity and the nature of motivated linguistic signs has remained marginal until at least the 1980s when interest in the topic increased particularly in functional and cognitive linguistics and more research started to appear (cf., among others, Haiman, 1980; Hamano, 1998; Simone, 1995). This trend continued and accelerated in the early 2000s (cf. Nielsen & Dingemanse, 2021).

Through most of the history of modern linguistics, the research on sound iconicity has focused on non-words, as exemplified by the bouba kiki effect (e.g., Ćwiek et al., 2022; Ramachandran & Hubbard, 2001). On the other hand, research on similar correspondences in real words has been limited and has only increased in the past 10 years, particularly in the area of ideophones. Ideophones are “marked words which depict sensory imagery” (Dingemanse, 2012). Their typological distribution is uneven, with three geographical clusters accounting for most of the languages (Africa, South America, South East Asia). The class of ideophones may be quite small or considerably large (hundreds or thousands of tokens). Ideophones, in contrast to onomatopoeia, depict a range of meanings such as movement, visual patterns, tactile perceptions, inner feelings, or cognitive states (Dingemanse, 2012). In various rating studies, that ask native speakers to evaluate words according to how much they sound like what they mean, ideophones are rated as more iconic than non-ideophones (Thompson et al., 2020). Ideophones are also phonologically marked, and they can, for instance, contain sequences of phonemes that do not conform to the phonotactic rules of the given language, as shown, for example, by Thompson et al. (2022) who studied the sequential probabilities in Cantonese ideophones and found these to be lower than those in non-ideophones, corroborating their phonemic salience.

These properties of ideophones have led to the question whether such words would be successfully paired with their meanings by language users who do not know the source language. Specifically, the phonemic and semantic salience of ideophones makes them prime candidates for testing whether language users can successfully infer meanings from words in an unfamiliar language. In a pioneering study, Dingemanse et al. (2016) used ideophones from five different languages as stimuli. Participants heard a word and had to select from two possible translations. Participants generally performed above chance level, with sound-mimetic words being the most successful. Similar results were replicated in subsequent studies (cf. Van Hoey et al., 2023 for a similar study with Cantonese speakers). McLean et al. (2023) used 304 Japanese words from the Japonic Sensory Lexicon (McLean, 2022), containing both ideophonic and prosaic words, and presented English speakers with recordings. They used a combination of a guessing task with iconicity rating. They modified the guessing task such that participants heard two Japanese recordings and decided which one is a more suitable equivalent for a single English word. They found a strong positive correlation between iconicity ratings and guesses. Interestingly, their participants were more successful when the target Japanese word and the distractor were as distant as possible in terms of their phonemic makeup which may be interpreted as highlighting the association between certain sounds and meanings.

Using a similar design, Diatka and Milička (2017) showed Czech speakers’ pairs of Hindi words in three conditions: two ideophones, one ideophone and one prosaic word, two prosaic words. They found that participants were more successful in identifying ideophones when paired with prosaic words, owing to their formal (phonemic) salience and depictive meanings. Thompson and Do (2019) propose that the iconicity of ideophones is afforded by speakers’ ability to create perceptuo-motor analogy and the accessibility of articulatory gestures. This makes their meaning more accessible. In a related study, Thompson et al. (2021) have shown on a sample of ideophones from 13 languages that some semantic features of ideophones are cross-linguistically associated with certain phonological features (e.g., friction is attracted to airflow sibilance).

However, non-ideophonic or non-onomatopoeic vocabulary has been shown to exhibit some degree of sound-meaning associations. For instance, high front vowels have been associated with smallness and back low vowels with largeness (e.g., Ohala, 1995; Winter & Perlman, 2021). Winter et al. (2022) found that trilled /r/ is associated with roughness across a number of languages. Johansson et al. (2020) show that sonority correlates with luminance and saturation in color vocabulary. Furthermore, typological surveys of large language samples have revealed that lexical items in the basic vocabulary also exhibit systematic associations between sound segments and meanings (Blasi et al., 2016; Erben Johansson et al., 2020). It has been also shown that words in particular languages that are similar in meaning also tend to be similar in form (Dautriche et al., 2017). This is in line with research on phonesthemes, phoneme sequences that are associated with particular meanings, such as English *gl-* which occurs frequently in words such as *glow* or *glitter*, denoting meanings connected to light (Bergen, 2004). There is also a substantial and growing body of literature that has focused on collecting iconicity ratings for large numbers of words (Hinojosa et al., 2021; McLean et al., 2023; Perlman et al., 2018; Thompson et al., 2020; Winter et al., 2017, 2024). These studies show that while high degree of iconicity is generally characteristic of onomatopoeia and ideophones, a number of prosaic words also tend to be rated as relatively iconic, suggesting that the presence of some residual or ad hoc iconicity is perceived in many areas of the vocabulary across languages. However, it should be noted that associations of the type discussed here need not necessarily be due to iconicity, but may rather constitute cases of systematicity, that is, regular statistical patterns (e.g., Dingemanse et al., 2015). For instance, while the magnitude iconicity of high front and low back vowels has been explained by the idea that small organisms tend to produce high pitch sounds (so called *Frequency code*, see Ohala, 1995), phonesthemes do not originate in motivated, sound-mimetic words, even though they tend to be rated as iconic (Winter et al., 2024). Similarly, there are common tendencies observed across languages, such as verbs tending to be longer than nouns and abstract nouns being longer than concrete nouns (Reilly et al., 2012). Words within the same word class often share a similar phonological profile, a feature that some researchers categorize as language-specific (Dingemanse et al., 2015), although others suggest there are cross-linguistic consistencies (de Varda & Strapparava, 2022). These regularities can be employed, consciously or subconsciously, by speakers to assess the meaning of unfamiliar words.

Instances of success in the word-matching task may also be attributed to the influence of *linguistic affinity*, which encompasses similarities due to genetic relationships or language contact. A foreign word might resemble a cognate or a related word in the speaker’s native language, as demonstrated by research on mutual intelligibility (e.g., Čéplö et al., 2016; Gooskens et al., 2018). We considered the influence of the affinity by asking Czech native speakers to guess the meanings of words from four languages with varying degrees of linguistic distance from Czech: Hindi, Japanese, Turkish, and Latvian. Latvian is a Baltic language geographically the closest to Czech, and there is a general consensus that Baltic and Slavic languages share certain features not found in other Indo-European languages (C. H. Brown et al., 2008). Gray and Atkinson (2003) date the divergence of Baltic and Slavic roots to approximately 3400 BP, while the divergence of the root leading to Hindi occurred around 6900 BP. Hindi, as another representative of Indo-European languages, shares some cognates with Czech. Liu et al. (2022) conducted a pairwise language distance analysis based on syntactic similarities, analyzing Parallel Universal Dependencies treebanks. Their sample included Czech, Hindi, Turkish, and Japanese. According to their measurements, Japanese differs the most from Czech, followed by Hindi and Turkish. Hindi, despite being an Indo-European language, is geographically more distant from Czech than Turkish, which is a Turkic language. Therefore, we would expect Latvian words to exhibit the highest resonance with Czech speakers, while Japanese may pose the greatest challenge for comprehension.

The experimental research concerned with the guessability of regular, prosaic words has been limited to an even smaller number of studies, compared to ideophones discussed earlier. However, such studies exploring the ability of speakers to guess meanings of words from unknown languages based just on their forms have occurred intermittently since as early as 1933 (e.g., R. W. Brown et al., 1955; Tsuru & Fries, 1933). The common denominator of these studies is that the stimuli used in the experiments are by and large based on pairs of antonyms, most typically dimensional adjectives. An exception to this is the study by Berlin (1995) who presented English speakers with a list of word pairs from Huambisa (Jivaroan, Peru). Each pair included one fish name and one bird name from the language. The participants were able to guess bird names with a success rate of 58%. In an early representative example, Kunihira (1971) used pairs of Japanese antonyms that were presented with translations to non-Japanese speakers. Kunihira found that participants performed above chance level regardless of whether the stimuli were presented with “expressive” or flat intonation or in writing, concluding that there was some degree of sound symbolic association between the words and their meanings. As an aside, it should be noted that sound symbolism may be used as an umbrella term that can cover both iconic phenomena and instances of systematicity. Nygaard et al. (2009) used pairs of Japanese antonym pairs from Kunihira (1971) to assess to what extent sound meaning associations facilitate vocabulary learning. Nygaard et al.’s participants learned Japanese words paired with (a) their actual meanings (e.g., BRIGHT—bright), (b) with their opposite meanings (BRIGHT—dark), and (c) with randomly selected words from their set of stimuli (BRIGHT—walk). They measured response latencies in a speeded choice task in which participants heard a single Japanese adjective and were shown two possible English translations from which they had to choose. They found that after three blocks of learning, participants reacted faster to the matching and opposite meanings compared to the randomly selected translations. Nygaard et al. concluded that their English participants were able to access the correspondences between particular meaning domains (e.g., brightness) and the forms of the Japanese words. Bankieris and Simner (2015) used a similar design in a study focused on the role of synesthesia in the perception of sound symbolism. They used four pairs of antonyms (big/small, bright/dark, up/down, loud/quiet) recorded by native speakers of 10 different languages. Their stimuli were presented in four blocks for the four semantic domains, and they asked participants to play a recording of an unknown word and to select its meaning from two given labels. Interestingly, they found that participants were able to guess the correct meaning above chance level only for the size domain.

In a recent follow-up, Hayakawa and Marian (2023) used a forced-choice antonym task to investigate whether there are systematic similarities between words with similar meanings in unrelated languages and whether such similarities predict guessability of these words. They used noun, verb, and adjective antonym pairs from nine languages (Japonic-Sino-Tai, Slavic, and Romance). Monolingual English speakers listened to a sequence of two words and were subsequently presented with two pairs of sequences of their English translations (e.g., *sharp: blunt* and *blunt: sharp*), and their task was to indicate which sequence corresponds to the sequence of the foreign words. They found that participants were able to guess the meanings above chance level. Furthermore, they performed better on stimuli from the Romance languages. Participants were also more successful when the phonological distance between the foreign words and their English counterparts was shorter.

Different variants of the forced choice task have become the de facto field standard. In a typical experiment, participants are presented with a pair of stimuli, and their task is to match the stimuli with meanings (be it depictions or translations into the participants’ language). Alternatively, a single stimulus is presented and participants pick from two possible translations. While participants have typically been relatively successful in such experiments both with ideophonic and non-ideophonic stimuli, some research suggests that this effect may be at least partially task-specific. For instance, Aveyard (2012) has found that when participants are given four choices instead of two, the iconicity effect is less pronounced (see also the discussion in Lockwood & Dingemanse, 2015; McLean et al., 2023).

The present study extends existing research on the guessability of word meanings by including words that cover a wide range of semantic domains and do not form opposites along a single dimension (as in Hayakawa & Marian, 2023 or Kunihira, 1971). The words were presented graphically using Czech orthography in quadruplets such that both foreign language stimuli could be presented simultaneously and prosody and other suprasegmental features were made unavailable. By employing this design, we studied the extent to which naïve speakers of Czech are able to connect pairs of words from different lexical classes and semantic domains to their translations. Our aim was to establish whether participants would perform similarly to the results of existing studies under these conditions and to investigate what strategies participants might employ when they cannot rely on phonemic (or semantic) salience—that is, when the words used were not ideophones and the word pairs were not, for example, antonyms. We decided to use words that come from a variety of different semantic domains to minimize possible implicit associations participants might build between recurring semantic features on the one hand and sounds/graphemes on the other. It was hypothesized that speakers will have the highest success rate in guessing the meanings in a close language, that is, Latvian, while Turkish and Japanese words will be matched with Czech translations less successfully.

We conducted a comprehensive analysis considering variables from various domains. These include measuring differences in length and semantic and phonological distances between the words presented in the trials. Phonological distances were measured using two methods—edit the phonological distance between a Czech word and its counterpart, and vector phonological distance using occurrence of distinctive phonetic features in the words (see more in detail below). In addition, we take into account their frequencies in corpora, their word classes, whether they are cognates, and various design-related features such as the order of trials and the presentation format of translations (whether in the same or reversed order as the Czech words). The contribution of the present study may be summarized as follows:

- The experimental setup was conducted across various languages (Latvian, Hindi, Turkish, and Japanese), allowing us to control for the influence of the language distance. The chosen languages deliberately exhibit varying degrees of linguistic relatedness and geographic proximity to Czech.
- The word selection was arbitrary, aiming to avoid representing variation solely along one dimension (such as in Bankieris & Simner, 2015). This approach enabled us to examine diverse characteristics of words that could potentially serve as cues for intelligibility.
- The pairs of words were assigned randomly and shuffled for each trial. Such design enabled us to uncover the effects caused by the interaction of the four words presented together in one trial. For example, a long Czech word and a short Czech word presented in one trial might have a better chance of being correctly assigned than two short Czech words presented together.
- The number of 1,128 Czech native speakers participated in the study. Such a large number enabled us to determine effect sizes and their associated confidence intervals for various anticipated effects, as described earlier.

## 2 Methodology

The protocol, scripts, datasets, and additional visualizations, including a detailed description of the methodology, are provided in Supplementary Material, which can be accessed under url: https://osf.io/fecq5 (mirrored on http://milicka.cz/kestazeni/IntelligibilityLaS2024.zip).

### 2.1 Materials

We employed the same set of Czech-Hindi word pairs used in the non-ideophonic word set introduced in Diatka and Milička (2017). These word pairs were selected randomly from a frequency wordlist derived from the Hindi corpus HindEnCorp (Bojar et al., 2014). The selection was based on the following criteria: 22 words from the lower-frequency level (with 1–13 occurrences in the corpus), 23 words from the middle-frequency level (with 14–200 occurrences in the corpus), and 23 words from the upper-frequency level (with over 200 occurrences in the corpus). These selected words were then translated into Turkish, Japanese, and Latvian. In cases where a word had multiple interpretations, one meaning was chosen at random. The words were presented to the participants in transliterated form, adhering to Czech orthographic rules. Czech orthography is a phonographic and phonemic writing system, which means that it allows for the pronunciation of unknown words to be closely approximated from the script itself (Beneš, 2013). The words and their translations were randomly assigned to pairs which were shuffled for each participant, therefore violating the recommendation stated by Ahlner and Zlatev (2010). The lists of words are presented in full length in the Appendix A.

### 2.2 Participants

The participants were highly proficient speakers of Czech. More than 95% of them were native Czech speakers, with a small percentage being native Slovak speakers who had resided in Czechia for an extended period and possessed near-native fluency in Czech. It is worth noting that Czech and Slovak are fully mutually intelligible languages, as indicated by, for example, Novotná and Blazek (2007) who suggest a similarity of 97%. As a result, these participants were included in our sample. None of the participants had any knowledge of the second language in the language pair (i.e., Hindi, Japanese, Turkish, or Latvian).

There were 1,128 participants in total, with 60.37% self-identifying as female, 39.63% as male, and 0% as non-binary. Average age was 23.53 years (for a detailed breakdown of the age distribution, see the Supplementary Material). The number of participants for each language pair was as follows:

- Turkish: n=540, average age 24.9, 63.9% female, 36.1% male, 0% non-binary;
- Japanese: n=254, average age 21.8, 57.09% female, 42.91% male, 0% non-binary;
- Hindi: n=164, average age 19.3, 53.05% female, 46.95% male, 0% non-binary;
- Latvian: n=170, average age 25.9, 61.18% female, 38.82% male, 0% non-binary.

The data for the Hindi group were collected from participants in the non-ideophonic word set group as reported in Diatka and Milička (2017). In that study, only the proportions of correct answers were calculated using this dataset.

All participants provided explicit consent for their participation in the experiment and for the scientific processing of the results, both verbally and through their interaction with the software used in the experiment. The data and demographic information were anonymized, and participants could easily withdraw from the study at any time by returning the tablet. The research received approval from the Ethical Committee of the Faculty of Arts, Charles University.

### 2.3 Procedure

The BlackSquare application (https://sourceforge.net/projects/blacksquare) was used for the experiment. It was carried out on Android tablets. Before the beginning of the experiment, the following instruction in Czech was displayed on the screen: “You are taking part in a linguistic experiment. Your responses will be used for scientific analysis. Each screen will display four words—two in Czech, which are translations of words from an unfamiliar language. Your task is to match these words correctly, even if you do not know the language. There are correct answers for this task, so rely on your language intuition.” Participants were required to tap the OK button to proceed.

**Figure 1.**
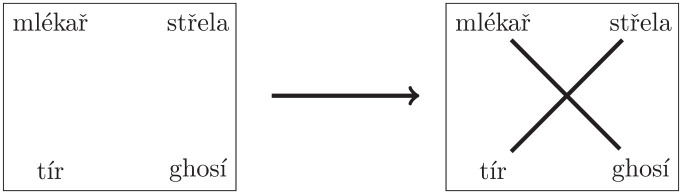
An example of an experiment trial. *Note.* The upper pair of words is in Czech (the first word means “milkman,” the second one means “shot”), and the lower pair is in Hindi.

Each participant received 34 assignments, each consisting of a set of four words: the upper pair in Czech and the lower pair in another language. The two Czech words had meanings corresponding to the two foreign words, with the order randomized. Participants were tasked with matching the corresponding words (see Figure 1). After completing the experiment, participants were informed about their results and asked to provide information about their age and gender.

The experiment was conducted in small groups of participants (up to 12) and was presented as a contest. The top three performers received prizes (e.g., candy or little snack). After the experiment was conducted, emphasis was placed on the role of luck in the experiment to prevent participants with lower scores from viewing their performance as a failure. One to two examiners were present throughout the experiment to ensure that participants did not cheat.

## 3 Results

Our analysis primarily focused on three aspects: the overall correctness of participants’ guesses across different languages, the correctness for individually words, and the influence of various variables on the correctness of these guesses. To evaluate the impact of independent variables on the likelihood of correct guessing, mixed-effects logistic regression was employed.

All data resulting from the experiments are available in the online Supplementary Material, where a detailed technical description of the analyses and the scripts used can also be found. Due to space constraints, it was not possible to focus on every variable individually beyond the mixed-effects model here. However, the Supplementary Material includes charts describing the demographic composition of the sample, the distribution of variables, the influence of various individual independent variables on average correctness, as well as other secondary dependent variables (e.g., log reaction time, number of corrections, etc.). The provided scripts are easily modifiable and can be used for the analysis of other variables that readers may be interested in exploring and for replication of the study on other languages.

### 3.1 Language pair

**Figure 2.**
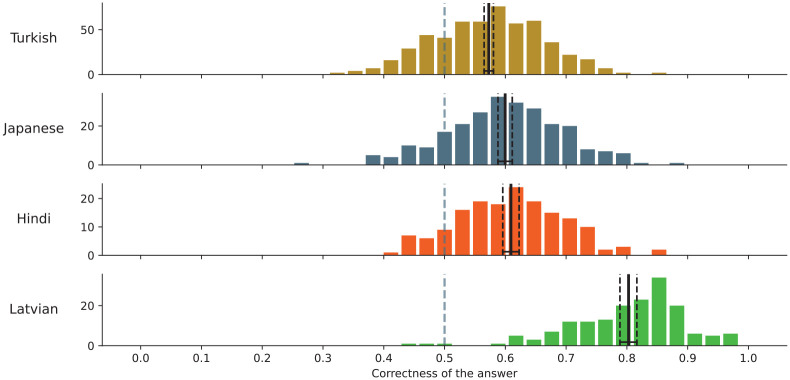
The distribution of the correctness of the answers.

Linguistic affinity appeared to significantly influence the correctness of answers (see Figure 2). Among our language pairs, the highest correctness was observed in Czech-Latvian (80.3%, the 95% paricipant-wise bootstrapped confidence interval [CI] is [78.8%, 81.7%]). This supports the hypothesis that Czech and Latvian, being the closest languages in our sample, would yield higher correctness. In contrast, the correctness rates in the other language pairs were comparatively lower and more consistent with each other: Czech-Hindi (60.9%, CI = [59.6%, 62.3%]), Czech-Turkish (57.3%, CI = [56.5%, 58.0%]), and Czech-Japanese (60.0%, CI = [58.8%, 61.2%]). Notably, the correctness rate for Czech-Japanese, despite the distance between these languages, did not substantially differ from the other pairs.

It should be noted that the success rate in Turkish translations may have been influenced by the method used for translating verbs from Czech. In contrast to other language pairs, the imperative forms of Turkish verbs were selected as translations, instead of the past and infinite verb forms in Czech. The motivation was to avoid an extreme systematicity of the Turkish agglutinative morphology in the verbal flexion which was assumed to overpower any other clues of intelligibility. However, this elimination of a systematic element, albeit in only five words, could have contributed to the lower success rate, since the impact of cues of systematicity might have been more pronounced had the verbs been translated into specific verb forms.

These findings suggest that a high degree of linguistic affinity, as seen in the Czech-Latvian pair, enhances performance. However, the baseline success rate appears to hover around 60%, irrespective of the degree of language relatedness.

### 3.2 Intelligibility of individual words

Upon examining the words used in the experiment, irrespective of their pairings, it was observed that certain words were more successfully guessed across all four languages, typically those with high frequency (e.g., *what*, *day*, *name*). Conversely, some words achieved success only in specific languages, often as cognates in related languages. This was particularly pronounced in Latvian, where 15 words had a guessing success rate exceeding 0.9. They were either cognates or closely resembled a Czech word from the same semantic domain (e.g., “kontakt” (ces)—“kontakc” (lav) *contact*, “země” (ces)—“zeme” (lav) *country*, “nehmotný” (ces)—“nemateriáls” (lav) *immaterial*). The influence of cognates on intelligibility was subsequently confirmed by the mixed model analysis (see Section 3.3).

However, even the words that were guessed with lower success rates exceeded, in the majority of cases, random guessing. With 68 words translated into four languages (resulting in 272 translations), only 22 were guessed below the 50% baseline. These instances could be ascribed to factors such as significant length asymmetry (e.g., “zaměstnanec” (cz)—“išči” (tur) *employee*). Conversely, 138 translations were guessed statistically significantly better than random choice.

In Appendix A, a comprehensive list of all Czech words and their translations in the tested languages is provided, along with the corresponding percentages indicating the accuracy of guesses and their binomial CIs (even more details to be found in the Supplementary Material).

### 3.3 Mixed models analysis

The results for individual words, however, do not offer the complete picture. Rather than focusing solely on individual word outcomes, it is more important to examine how a word performs in combination with another word in the word pair. Because the combinations were random, we can investigate numerous hypotheses concerning the relationships between words. For example, if a Czech word is short but its translation is long, and it is combined with another word that is long but whose translation is short, we might hypothesize that participants will fail to guess this pair correctly due to confusion arising from word-length disagreement. We examined several variables we considered potentially important: the number of cognate words in the pair; whether the two words belong to the same part-of-speech class; length agreement (as outlined earlier); frequency agreement; semantic distance between the words in the pair; and vector and edit phonological similarity between a word and its translation.

Detailed descriptions of these variables, their operationalizations, and results can be found in the respective sections below. In addition, we included technical variables related to data collection: inverse trial, indicating whether the word pair was presented to the participant in the correct or reversed order, and trial order, as it may matter whether the word pair was presented at the beginning of the session (when the participant was fresh but inexperienced) or toward the end (when they might have become fatigued). Participant demographic variables (age and gender) were also included. All these factors were integrated into a mixed-effects model.

Given that the target variable was binary (participants could either answer correctly or incorrectly for each word pair), we employed mixed-effects logistic regression. This statistical approach is particularly suitable for data where observations are not independent, since multiple responses come from the same participant. Mixed-effects logistic regression also allowed us to analyze participant-specific independent variables (gender and age).

The random components included *Cognate*, *Part of speech mismatch*, *Semantic distance*, *Edit phonological similarity*, *Vector phonological similarity*, *Length agreement*, *Frequency (log) agreement*, *Inverse trial*, and *Trial order*, that is, all variables except *Gender* and *Age*, which are participant-specific. All covariates were standardized. Model details along with summary statistics for each language are available in Appendix B.

The analysis was conducted separately for each language to prevent artifacts arising from the mixing of data sets. This approach was particularly pertinent given the distinct participant demographics, the variable number of participants across different languages, and the varying number of variables considered for each language (it was not logical to track the influence of cognates in unrelated language pairs, such as Czech-Turkish and Czech-Japanese). For other variables, if they were not tested across all languages, it is more accurate to discuss these cases in terms of a failure to replicate the effect, rather than attributing it to language specificity.

**Figure 3.**
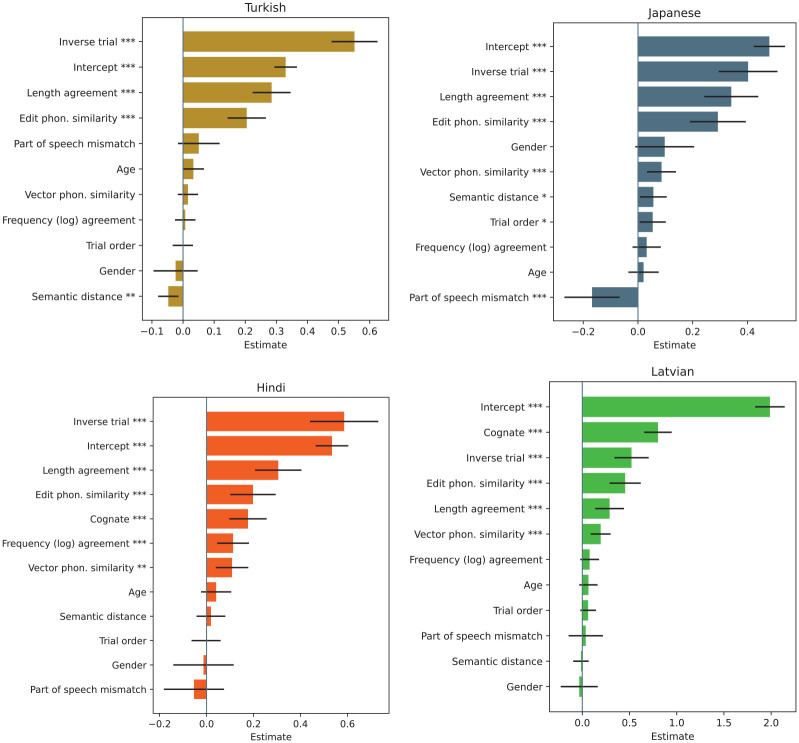
Mixed model coefficients of independent variables. *p <.5; **p <.1; ***p <.0001.

As can be seen in Figure 3, four variables exhibited a statistically significant influence on intelligibility across all languages. We will comment on these variables first. Subsequently, we will address the remaining variables that either lacked significance or demonstrated significance only in some languages.

#### 3.3.1 Cognates

The presence of cognates was operationalized as a binary variable, with some partial non-binary values, typically when only one of several morphemes was a cognate. For example, *din* (Hindi)—*den* (Czech)—“day” and *kjá* (Hindi)—*co* (Czech)—“what” were (as full cognates) coded as 1, while *kučh* (Hindi)—*něco* (Czech)—“something” was coded as a partial cognate (0.5). The overall score for the word pair is calculated as an addition of scores for each word.

The Latvian cognates (e.g., *plānots* (Latvian)—*plánovaný* (Czech)—“planned,” or *sniegs* (Latvian)—*sníh* (Czech)—“snow”) were coded by Michal Škrabal, and the Hindi cognates by Jan Bičovský (non-author, expert in diachrony of Indo-European languages).

As mentioned before, the presence of cognate words between the Czech language and its translation was found to significantly enhance the correctness of guessing the word pair. This variable was relevant only for Hindi and Latvian, as the Turkish and Japanese word lists did not include such words.

The effect was notably more pronounced in Latvian, with a log odds ratio estimate of 0.801 (95% CI = [0.66, 0.95], *p* <.001), than in Hindi, which had a log odds ratio estimate of 0.18 (CI = [0.10, 0.26], *p* <.001).

This finding aligns with expectations, particularly for Latvian, where cognates are relatively easy to identify, even for non-linguists. What is more intriguing is the observed effect in Hindi, considering the vast temporal gap that separates modern Hindi and Czech from their common ancestral language.

#### 3.3.2 Length agreement

Short words in one language often have short equivalents in other languages, that is, there is a positive cross-linguistic correlation in word length. Participants may have some access to this and rely on similarity in word length. Furthermore, there is evidence that semantic complexity is correlated with word length (Lewis & Frank, 2016), which can again be exploited by participants based on the assumption that semantic complexity of words is similar across languages. Our word list, randomly selected from a corpus, is no exception. The length of words in our list in Czech correlates with their lengths in Latvian ρ=0.67, Hindi ρ=0.45, Japanese ρ=0.46, and Turkish ρ=0.41. This corresponds to the assumption that both words carry approximately the same amount of information and that redundancy is allocated roughly proportionally.

When word pairs display noticeable length differences in *both* languages, these discrepancies can serve as cues for participants. For instance, the word pairs “den” (Czech)—“gün” (Turkish) meaning *day* and “znepokojení” (Czech)—“endişe” (Turkish) meaning *unease (noun)* can be relatively easily guessed correctly by matching the longer Czech word with its longer Turkish counterpart. Conversely, in the case of the *milkman*—*shot* pair (refer to Figure 1), where the lengths of the Czech words are identical (each comprising six phonemes), length does not provide any useful guidance.

Therefore, we introduce a metric called *Length Agreement* (LA), which quantifies the difference in length between the two words in both languages and then calculate the geometric mean of these differences. This approach ensures that a zero distance is preserved if the words are of the same length in one or both languages (and thus the length difference can conway no information for the participant). Sometimes, a pair may consist of a short Czech word translated into a relatively long word, and vice versa. In such cases, we assign a negative value to the LA metric to reflect that this configuration might be confusing for the participant. The formula for LA is as follows:

(1) $$\mathrm{LA}\left(C_{1},C_{2},T_{1},T_{2}\right)=\mathrm{sign}\left(\left(C_{1}-C_{2}\right)\left(T_{1}-T_{2}\right)\right)\sqrt{\left|\left(C_{1}-C_{2}\right)\left(T_{1}-T_{2}\right)\right|}.$$

There are numerous ways to measure word length (number of phonemes, syllables, etc.). However, since these variables are interrelated, we chose a single operationalization: the number of phonemes. This is also the metric referred to in subsequent sections when discussing other length-related variables.

**Figure 4.**
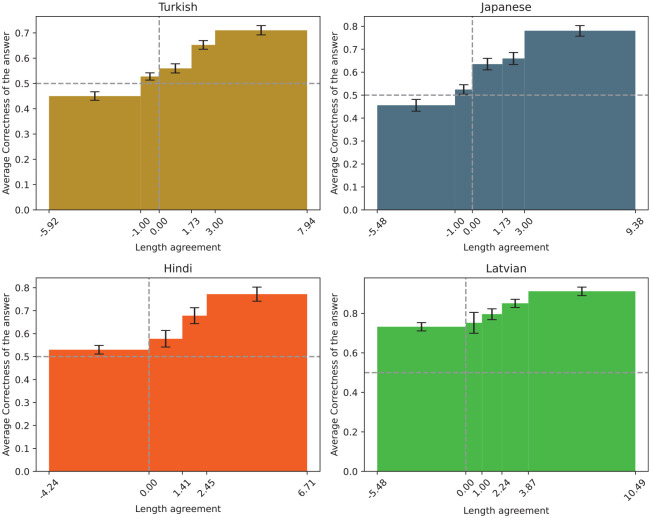
The correctness of the answers in respect to length agreement.

The LA metric was found to have a statistically significant positive overall effect on the correctness of answers (*p* <.001 in all four languages, see Figure 4), namely the log odds likelihood is 0.28 for Turkish (CI = [0.22, 0.35]), 0.34 for Japanese (CI = [0.24, 0.43]), 0.31 for Hindi (CI = [0.21, 0.40]), and 0.30 for Latvian (CI = [0.14, 0.44]). This finding supports the hypothesis that participants possessed either an explicit or intuitive understanding of language communication efficiency rules, which facilitated their ability to guess the meanings of words.

#### 3.3.3 Edit phonological similarity

It can be hypothesized that participants tend to match words that are phonologically similar, seeking mutual resemblances even in languages known to be distant and dissimilar. This hypothesis was corroborated using a variable we named *Edit phonological similarity*.

This metric is based on edit distance between Czech words and their counterparts. The edit distance metric utilized International Phonetic Alphabet (IPA) transcriptions, considering not only the number of phoneme changes but also the number of changed distinctive features. For this, the function *weighted_feature_edit_distance()* from the *panphon* package by Mortensen et al. (2016) was used. The metric is based on the sum of distances between the Czech word and its translation (Dist(C1,T1)) from which the distances of the non-matching counterparts (Dist(C1,T2)) $\left(Dist\left(C_{1},T_{2}\right)\right)$ are subtracted. Subsequently, the sign is inverted, as distance is the inverse of similarity:

(2) $$\mathrm{PS}\left(C_{1},C_{2},T_{1},T_{2}\right)=\mathrm{Dist}\left(C_{1},T_{2}\right)+\mathrm{Dist}\left(C_{2},T_{1}\right)-\mathrm{Dist}\left(C_{1},T_{1}\right)-\mathrm{Dist}\left(C_{2},T_{2}\right).$$

**Figure 5.**
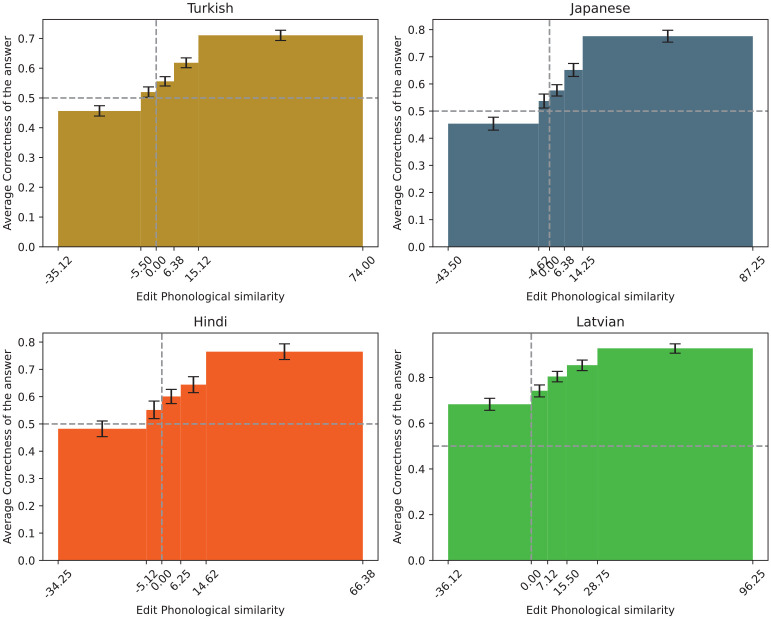
The correctness of the answers in respect to edit phonological similarity.

The edit phonological similarity between Czech and its translations was found to have a significant effect on the correctness of the answers across the languages. Specifically, the log odds likelihood is 0.20 for Turkish (CI = [0.14, 0.27]), 0.29 for Japanese (CI = [0.19, 0.39]), 0.20 for Hindi (CI = [0.10, 0.29]), and 0.45 for Latvian (CI = [0.29, 0.62]). This indicates that the greater the phonological similarity between the Czech word and its translation, the greater the likelihood of a correct guess. This tendency is particularly notable in Latvian, where phonological similarity often indicates semantic proximity, a consequence of the linguistic affinity between the languages (see Figure 5).

#### 3.3.4 Vector phonological similarity

It should be noted that while edit phonological similarity was a significant predictor of increased guessability, the distances between the Czech words and the unknown words are on the whole relatively large. Median distance between the unknown words and the Czech targets was over 20 for all the languages. For instance, the median distance for the Japanese set was 23.2. A pair such as *směr*—*hōkō* “direction” has a distance of 23.5. Importantly, the median of the median distances between the individual Czech words and all of the unknown words was very similar, ranging from 21.8 for the Hindi set to 27.2 for Latvian, meaning that the unknown words and their targets were on the whole quite dissimilar. Thus, the point may be made that overall the distances or similarities between the Czech words and their foreign counterparts were relatively large, the participants could still have used even very small similarities such as the presence of a round vowel. This is due to the fact that the metric employs edit distance on phonological features such that, for example, Czech *den* and Hindi *din* “day” have a distance of 0.5 owing to the fact that they differ in a single phonological feature; on the other hand, the latvian counterpart *djena* has a distance of 14.5 from its Czech counterpart which is considerably higher. In fact, the distance provided by the metric used is greater than between *den* and its Japanese equivalent *hi* (11.875). However, it is safe to assume that Czech speakers will have no trouble to identify the Latvian word as the correct equivalent of the Czech target (the words were paired correctly on almost 93% of trials, whereas the Japanese equivalent was correctly identified only on 71% of trials). While this is in part accounted for by the cognacy variable, there is nonetheless a possibility that words that appear to be quite dissimilar in terms of the metric used are quite alike for the participants.

To check for this, we have devised a complementary metric which we believe is closer to how language users may perceive similarity, since it relies more on the overlap of sounds and their features in the stimulus material. This was done by describing the individual sounds in the Czech words and their counterparts in terms of phonological features. Twelve features in total were used, based on Thompson et al. (2021), who investigated cross-linguistically recurring co-occurrences of phonological features and semantic domains in ideophones. We then calculate Euclidean distances in a twelve-dimensional space defined by these features. We refer to the similarity metric derived from these distances as *vector phonological similarity*, which is also calculated according to formula 2, just edit distance is replaced by euclidean distance of vectors. Using this new metric, the distances between Czech words and their foreign counterparts change noticeably. For instance, the distance between *den* and the Latvian *djena* is 0.4055, while the distance for the Japanese *hi* is 1.1667. It is thus unsurprising that these two phonological similarity metrics do not correlate strongly with each other: the highest correlation is in Latvian ( ρ=0.68 ), close to zero in Hindi ( ρ=0.03 ) and in Turkish ( ρ=−0.16 ), and negative in Japanese ( ρ=−0.75 ).

When entered as an additional predictor in the model, this new metric is also a significant predictor of correctness for all the languages with the exception of Turkish—specifically, the log odds likelihood is 0.02 for Turkish (CI = [−0.02, 0.05]), 0.09 for Japanese (CI = [0.03, 0.14]), 0.11 for Hindi (CI = [0.04, 0.18]), and 0.19 for Latvian (CI = [0.09, 0.30]).

**Figure 6.**
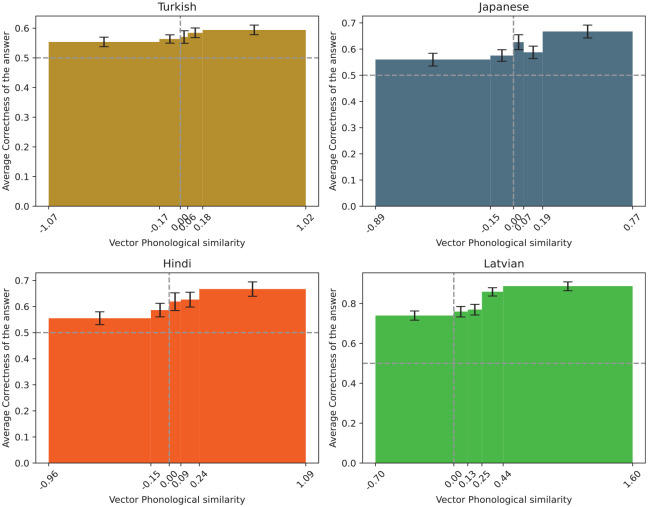
The correctness of the answers in respect to vector phonological similarity.

Pairs in which the corresponding Czech and foreign word were more similar than the other Czech word and its counterpart were guessed more successfully. Interestingly, the effect of this metric was smaller for all the languages than the edit phonological similarity, suggesting that the contribution of overlapping features to the guessability was relatively small, which is also visible in Figure 6.

#### 3.3.5 Inverse trial

**Figure 7.**
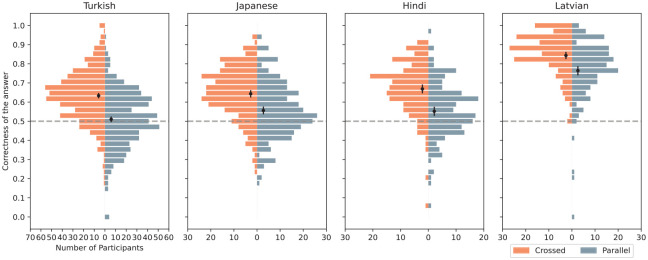
The distribution of the inversed answers, the average, and 95% confidence intervals.

In the tasks presented to participants, the order of translations could be reversed, meaning that the correct answer required participants to link words either parallel (vertically) or diagonally across the given options. Although there was an equal distribution of translations being and not being shown diagonally, participants were observed to prefer the diagonal pairing more frequently than the vertical one, as depicted in Figure 7.

This implies that whenever a word pair was presented diagonally, participants were more likely to choose the correct pairing. This tendency is reflected in our mixed model analysis—the *Inverse trial* binary variable exhibits a strong and statistically significant effect on correctness in all four languages, and the log odds likelihood is 0.55 for Turkish (CI = [0.48, 0.62]), 0.40 for Japanese (CI = [0.29, 0.50]), 0.59 for Hindi (CI = [0.44, 0.73]), and 0.52 for Latvian (CI = [0.34, 0.70]). Note the overlapping CIs, which are consistent with the assumption that the effect is not language-dependent given that the conditions of the experiment were comparable across all languages.

This observation highlights the importance of randomization and balanced design in experimental settings.

#### 3.3.6 Variables without an overall significant effect

The other variables in our study were found to have no overall effect on the correctness of the answers. However, some of them were found significant when looking at the individual languages. We will comment on each of them.

**Frequency (log) agreement.** Unsurprisingly, the word frequencies measured in the Czech corpus (Křen et al., 2020) correlate with the frequencies of their translations in the corpora of the respective languages, with Pearson’s Correlation Coefficient of ρ=0.84 for Latvian, ρ=0.72 for Hindi, ρ=0.55 for Japanese, and ρ=0.59 for Turkish. These correlation values are even higher than those for word lengths.

This raises the question of whether participants made decisions based on word length, or also based on other word properties that correlate with frequency, such as word length combined with phonotactic complexity and other factors. We have operationalized frequency agreement using the same formula as LA (Equation 1), substituting logarithms of frequencies for lengths. The lengths are logarithmized, since the lenght–frequency relation is exponential (Zipf’s law of abbreviation, see Bentz & Ferrer Cancho, 2016; G. K. Zipf, 1949).

However, this variable yields poorer results than LA (with a statistically significant effect only for Hindi, although the influence in other languages is also positive).

**Semantic distance**. The hypothesis tested by this variable posits that when the two words presented in the pair are semantically dissimilar, this semantic disparity should correlate with numerous differences in form; hence, semantically distinct words should be easier to match with their translations. We determined the semantic distance for each word pair (namely, the Euclidean distance of their Czechembeddings, as derived from Fares et al., 2017). However, it appears that the success rate is virtually independent of this variable. A statistically significant dependency was found only in Turkish and Japanese, but the direction of the effect in these languages is opposite and minor (with the log likelihood estimate being −0.05 for Turkish and 0.06 for Japanese). In both cases, the CI is only about 0.01 away from zero.

**Trial order.** This variable indicates the sequence in which the word pairs appeared in the study. Predicting its impact is challenging, as on one hand, participants can gradually develop a better intuition for foreign word forms as they encounter more examples, but on the other hand, they may become desensitized by fatigue and diminishing motivation. Statistical significance was attested only for Japanese, but even there, the effect size estimate was quite low at 0.05, with the lower limit of the CI nearly touching zero (0.006–0.10). This could mean either that the variable has no influence on success rates or that the two aforementioned opposing effects associated with this variable may have neutralized each other.

**Part of speech mismatch.** This metric assesses whether the two words presented in one trial belong to the same or different word classes. The analysis revealed no significant effect of this variable, indicating that participants were not able to leverage the morphological structure of the foreign language.

**Age and gender.** In addition to the characteristics of the words and word pairs, we incorporated participants’ age and gender as independent variables. Our analysis revealed no significant effects of these variables in any of the languages studied.

#### 3.3.7 Residual correctness

We aim here to determine whether our variables accounted for all factors influencing participants’ accuracy, or if there remained room for additional important unexplored clues aiding participants in identifying correct answers. Specifically, we are interested in whether participants could correctly pair words better than chance when words were not cognates, had no clues regarding length or frequency agreement, exhibited neither edit nor vector phonological similarity, and when the semantic distance between words was average.

This can be calculated from the model’s intercept, which represents these baseline odds.

However, since our model employs normalized values of the variables (each variable have different scales), the intercept reflects the probability of participants responding correctly when all variables are at their average values. This is not our interest; we need the scenario at zero values, not average.

Thus, to ascertain the baseline odds accurately, we re-ran the model without normalization and without demographic and technical variables (trial order, inverse trial). Only continuous variables remained: *Cognate*, *Semantic distance*, *Edit phonological similarity*, *Vector phonological similarity*, LA (without normalization or centering), and *Frequency (log) agreement* (centered).

The resulting baseline accuracies were:

- Turkish: 53.1% (95% CI = [52.2%, 54.1%])
- Japanese: 54.3% (95% CI = [53.0%, 55.8%])
- Hindi: 53.1% (95% CI = [51.2%, 55.0%])
- Latvian: 62.9% (95% CI = [69.9%, 65.8%])

These results mean there is still some space for additional factors in analysis of all four languages, but it is quite small (except for Latvian, where the baseline correctness is quite large).

## 4 Discussion

In our large-scale exploratory study, we aimed to identify the key cues enabling participants to infer the meaning of words in an unfamiliar language, distinct and distant from their mother tongue (Czech): Latvian, a Baltic language that has some affinity with Slavic languages and is within a common geographical area with Czech (C. H. Brown et al., 2008; Gray & Atkinson, 2003); Hindi, an Indo-European language that shares some cognates with Czech; Turkish, a Turkic language that does not have common root with Czech but is geografically fairly close; and Japenese, that has no common developmental root with Czech, and is geografically the most distant. This research encompassed various independent variables related to phonology, semantics, word length, and frequency, in addition to the participant’s age and gender.

Our findings revealed several variables with significant effects across the four languages used in the experiment. Crucially, participants were able to assign meanings to randomly selected pairs of words from a corpus more accurately than chance, even in languages unrelated to their own. This outcome allows us to assert that languages typically considered entirely incomprehensible can still convey non-zero information. This insight is particularly relevant for tests of mutual intelligibility of languages that have been traditionally focused on languages of closer affinity (Čéplö et al., 2016; Gooskens et al., 2018). Our finding demonstrates that zero mutual intelligibility should not be the default null hypothesis worth falsification attempts.

Nevertheless, our study found that participants performed better with a language traditionally considered closer to Czech than the others. Latvian yielded a success rate of 80.3% (95% CI = [78.8%, 81.7%]). In contrast, the other three languages showed lower success rate: Turkish at 57.3% (CI = [56.5%, 58.0%]), Japanese at 60.0% (CI = [58.8%, 61.2%]), and Hindi at 60.9% (CI = [59.6%, 62.3%]).

Furthermore, cognates played a significant role. Participants were better at identifying words that were cognates, not only in Latvian (log odds likelihood estimate 0.80, CI = [0.66, 0.95]) but also in Hindi (0.17, CI = [0.10, 0.26]), which is only loosely related to Czech through a shared Proto-Indo-European heritage. However, even the more distant affinity did not diminish the impact that this clue had on connecting the word with its translation in the matching task.

Overall, some words were matched more accurately than others (see the Supplementary Material). However, during the mixed-effect model analysis, our focus was not on individual words but rather on their pairwise relationships.

It was observed that participants tended to look for phonological similarities even in languages unrelated to Czech. When Czech words in the pair had some phonological resemblance to their foreign counterparts, it positively influenced the success rate in guessing (variable named *Edit phonological similarity*, *p* <.001 in all four languages, *Vector phonological similarity*, *p* <.001 in all four languages).

Another variable influencing overall success across all languages was LA. Participants were more successful when a long Czech word corresponded to a long word in the foreign language, and vice versa. Conversely, when there was a misalignment in word length—a long word in the foreign language corresponding to a short Czech word—it confused participants, leading to less accurate guesses. This finding indicates that people can utilize, whether consciously or not, the length of words as a cue in language comprehension. It aligns with the conclusion of Bankieris and Simner (2015) that speakers are helped by their intuition about systematic regularities in language. The finding that frequent short words like *day* or *name* had an overall high success guessing rate in all their translations suggests that speakers are able to utilize an intuition about information theory (Levshina, 2022) and Zipf’s laws (G. Zipf, 1936) and use it as a cue for the matching task.

Given that word lengths and frequencies are correlated according to Zipf’s Brevity Law (Corral & Serra, 2020), we expected to find an analogical effect in this measurement, that is, when the word frequency of the Czech word and its translation is close, it will increase a chance of correct matching. However, this hypothesis was not confirmed consistently.

A similar inconsistency was found when examining semantic distance: the degree to which words are semantically different had only a minor effect in Turkish and Japanese, with small and opposing effect sizes and CIs close to zero. In their study, Reilly et al. (2012) investigated the speakers’ ability to infer meanings of abstract and concrete nouns. They concluded that the semantic profile helps in guessing the meaning only when it aligns with the assumption that concrete nouns are short, while abstract nouns are lengthy and complex. Similarly, in our research, we did not observe a consistent impact solely attributable to semantic distance.

Regarding variables related to the experiment design, one notable aspect was the order in which the translations were presented. Participants tended to match word pairs in inversed order, preferring to make a diagonal instead of parallel vertical connections. Consequently, pairs presented in a mismatched order were guessed correctly with higher probability. This unexpected finding does not lend itself to any direct linguistic conclusions, but it underscores the importance of randomization and shuffling of trials in experiment design, as well as the balance in item presentation. Conversely, the number of trials already completed by participants did not have a significant effect, indicating that participants maintained consistent performance throughout the experiment. This result suggests that there was no progressive learning effect where participants became better over time, nor was there a fatigue effect leading to poorer performance toward the end of the experiment, or that they canceled out each other.

Interestingly, the model shows that even when there is no difference in lengths, phonological distances, or cognacy, participants did, on the whole, perform above chance level for all the languages. This suggests that there is some residual level of “associability” that may be due to iconicity or systematicity not accounted for by the predictor variables. This might suggest that some associations exist between the words in our sample similar to what Thompson et al. (2021) have shown for ideophones or Blasi et al. (2016) for the words of basic vocabulary.

An interesting framing for this finding may be found in Ahlner and Zlatev (2010) who discuss iconicity in language within the framework of cognitive semiotics. The type of guessing experiment used in our study as well as in the research discussed above is part of their discussion. They propose a model according to which the design of the task (as in, e.g., Nygaard et al., 2009) helps establish a higher-order analogy between two composite analogous grounds. This is due to the fact that the unknown words are antonyms that always contrast on a single dimension (e.g., big vs. small and SIZE or young vs. old in AGE, etc.) and the fact that translations in the participants’ language (synonymous linguistic signs) are provided. Our results show an above-chance ability to match even pairs of randomly selected words that do not necessarily form a meaningful contrast in any particular quality dimension, indicating that there is some residual iconicity or systematicity on top of the effects of similarity in phonological form or length or indeed cognacy. Simultaneously, this shows that not all the conditions assumed by Ahlner and Zlatev (2010) need always be fulfilled in order for participants to correctly guess the meanings of unknown words. Our findings thus also tie in with the results of McLean et al. (2023) whose design also diverts from the set up discussed by Ahlner and Zlatev.

Finally, the ability to provide correct answers appears to be evenly distributed across the population, as indicated by the lack of significant influence from age or gender. The participants performed with equal success rates regardless of gender. While some participants’ features, such as dyslexia (Drijvers et al., 2015) or synaesthesia (Bankieris & Simner, 2015), were found to influence the ability to guess meanings, age and gender seem to not be one of these.

## Figures and Tables

**Figure A1.**
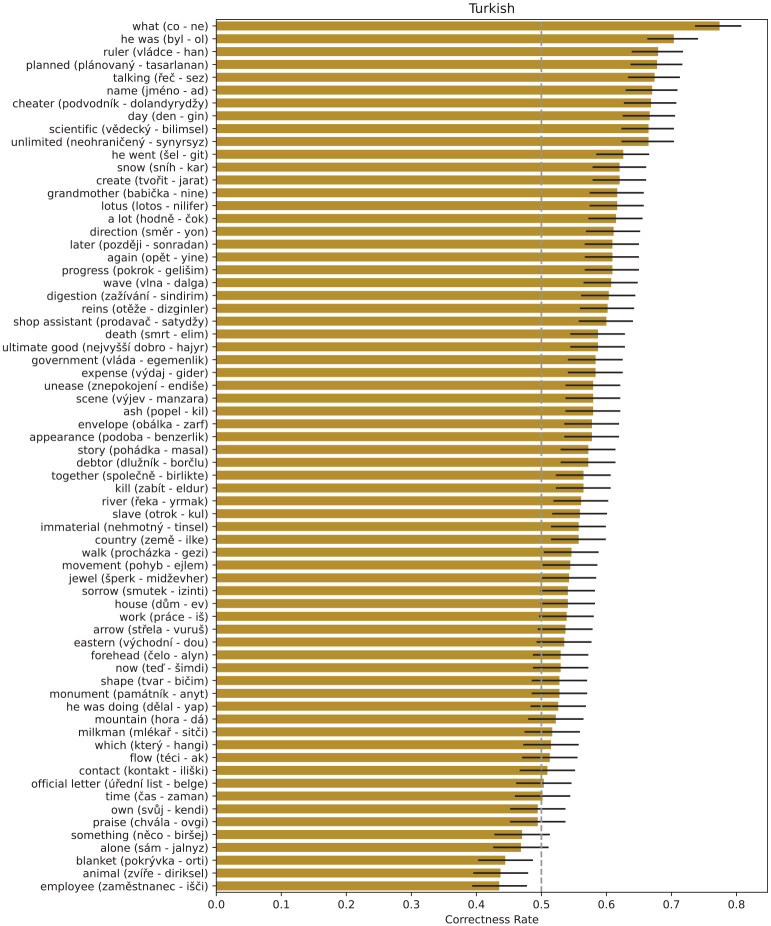
List of words translated to Czech and Turkish (Czech transcription). Correctness of individual words with their 95% confidence intervals (bootstrap resampling).

**Figure A2.**
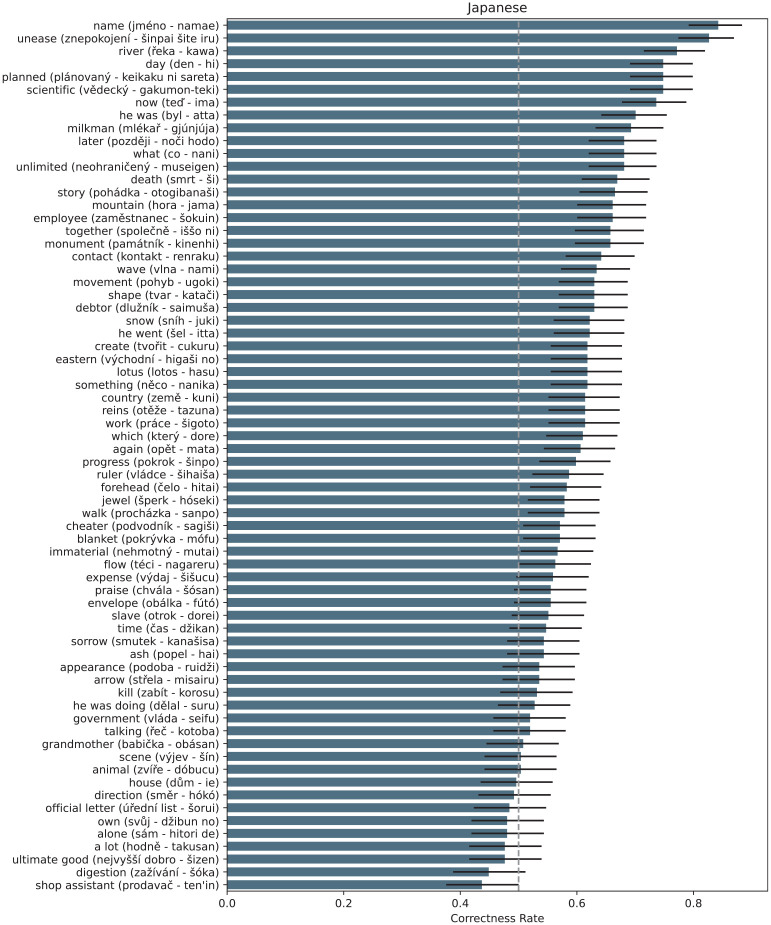
List of words translated to Czech and Japanese (Czech transcription). Correctness of individual words with their 95% confidence intervals (bootstrap resampling).

**Figure A3.**
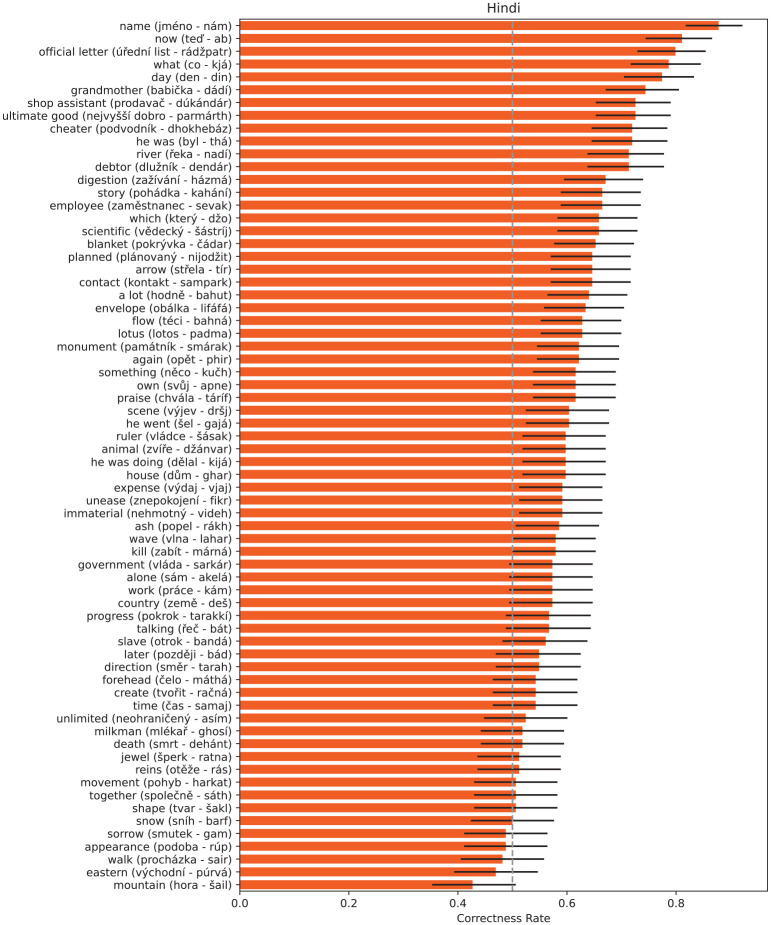
List of words translated to Czech and Hindi (Czech transcription). Correctness of individual words with their 95% confidence intervals (bootstrap resampling).

**Figure A4.**
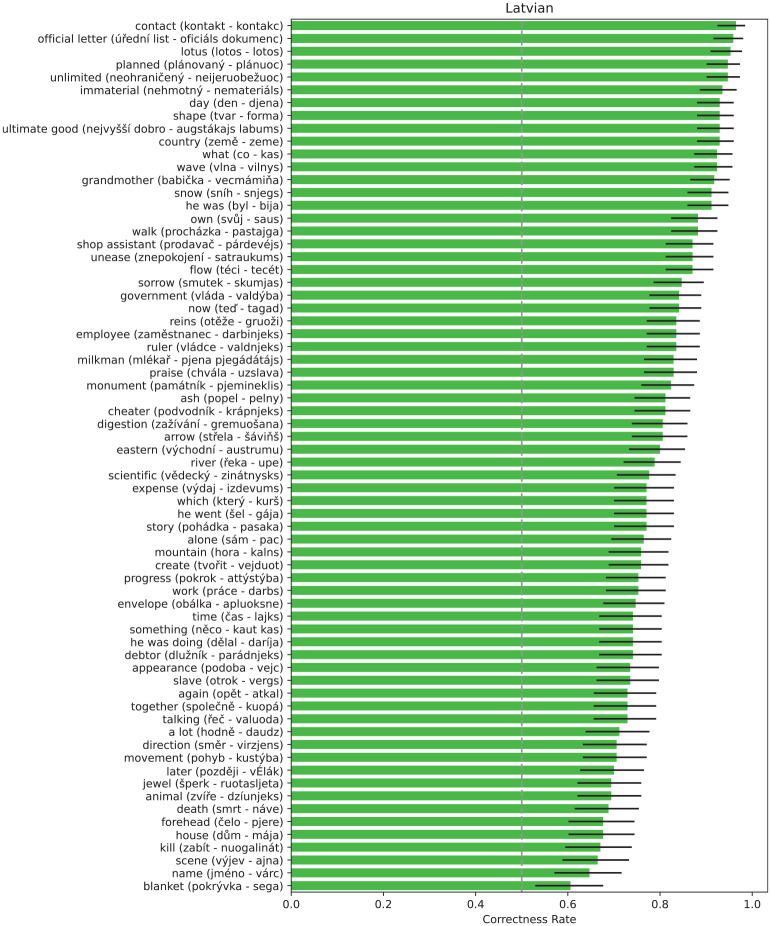
List of words translated to Czech and Latvian (Czech transcription). Correctness of individual words with their 95% confidence intervals (bootstrap resampling).

## Appendix B

### Mixed-effects model

**Table B1.** Model Specification.

Call	glm: Correctness of the answer ∼ 1 + Gender + Part of speech mismatch + Inverse trial + Question order + Frequency (log) agreement + Length alignment + Semantic distance + Age + Vector phon. similarity + Edit phon. similarity + (1 + Part of speech mismatch + Inverse trial + Trial order + Frequency (log) agreement + Length agreement + Semantic distance + Vector phon. similarity + Edit phon. similarity—Unique ID)	
Description	Value	Comment
Model type	Logistic	Model for binary y
Optimizer	bobyqa	
Link function	Logit	Log of the odd of y = 1 over y = 0
Direction	P(y = 1)/P(y = 0)	$\frac{P\left(Correctness\;of\;the\;answer=1\right)}{P\left(Correctness\;of\;the\;answer=0\right)}$
Distribution	Binomial	Dichotomous event dist. of y

**Table B2.** Overall comparison across languages.

	Turkish	Japanese	Hindi	Latvian	Comment
LogLikel.	−11944.739	−5452.21	−3490.44	−2487.45	Unconditional log-likelihood
-2*LogLikel.	23889.48	10904.43	6980.88	4974.9	Unconditional abs. deviance
Deviance	22656.589	10268.69	6558.279	4403.74	Conditional relative deviance
R-squared	0.078	0.114	0.113	0.274	Marginal
R-squared	0.1372	0.181	0.185	0.442	Conditional
AIC	24001.5	11016.4	7114.9	5108.9	Akaike information criterion
BIC	24439.28	11412	7558.8	5555.3	Bayesian information criterion
Residual DF	18304	8580	5509	5713	
χ^2^/DF	0.9295	0.918	0.918	0.762	Overdispersion indicator
Converged	Yes	Yes	Yes	Yes	

**Table B3.** Detailed results—Turkish.

			95% Conf. Int.			95% Conf. Int.			
			Estimate			Exp(B)			
Variable	Estimate	*SE*	Lower	Upper	Exp(B)	Lower	Upper	z	*p*
Intercept	0.32978	0.0185	0.2935	0.3661	1.391	1.341	1.442	17.8168	<.001
Gender	−0.02392	0.0361	−0.0948	0.0469	0.976	0.91	1.048	−0.6619	.508
Part of speech mismatch	0.05065	0.0342	−0.0163	0.1176	1.052	0.984	1.125	1.4829	.138
Inverse trial	0.55117	0.0377	0.4773	0.6251	1.735	1.612	1.868	14.6181	<.001
Trial order	−0.000686	0.0166	−0.0332	0.0318	0.999	0.967	1.032	−0.0413	.967
Frequency (log) agreement	0.00707	0.0168	−0.0258	0.04	1.007	0.975	1.041	0.4213	.674
Length agreement	0.2848	0.0311	0.2239	0.3457	1.329	1.251	1.413	9.1613	<.001
Semantic distance	−0.04739	0.0166	−0.0799	−0.0148	0.954	0.923	0.985	−2.8534	.004
Age	0.03317	0.0174	−0.0009	0.0672	1.034	0.999	1.07	1.9102	.056
Vector phon. similarity	0.01578	0.0166	−0.0168	0.0484	1.016	0.983	1.05	0.9492	.343
Edit phon. similarity	0.20474	0.0314	0.1432	0.2663	1.227	1.154	1.305	6.5205	<.001

**Table B4.** Detailed results—Japanese.

			95% Conf. Int.			95% Conf. Int.			
			Estimate			Exp(B)			
Variable	Estimate	*SE*	Lower	Upper	Exp(B)	Lower	Upper	z	*p*
Intercept	0.4803	0.0291	0.42326	0.5374	1.617	1.527	1.711	16.501	<.001
Gender	0.0978	0.0549	−0.00979	0.2053	1.103	0.99	1.228	1.782	.075
Part of speech mismatch	−0.1678	0.0515	−0.2688	−0.0669	0.845	0.764	0.935	−3.258	.001
Inverse trial	0.4026	0.0548	0.29514	0.51	1.496	1.343	1.665	7.345	<.001
Trial order	0.0539	0.0244	0.00608	0.1016	1.055	1.006	1.107	2.21	.027
Frequency (log) agreement	0.0318	0.0264	−0.01987	0.0835	1.032	0.98	1.087	1.207	.227
Length agreement	0.3411	0.0504	0.24239	0.4398	1.407	1.274	1.552	6.773	<.001
Edit phon. similarity	0.292	0.0522	0.18963	0.3944	1.339	1.209	1.483	5.59	<.001
Semantic distance	0.0564	0.0248	0.00775	0.1051	1.058	1.008	1.111	2.272	.023
Age	0.0204	0.0283	−0.03501	0.0759	1.021	0.966	1.079	0.722	.47
Vector phon. similarity	0.0862	0.027	0.03323	0.1392	1.09	1.034	1.149	3.189	.001

**Table B5.** Detailed results—Hindi.

			95% Conf. Int.			95% Conf. Int.			
			Estimate			Exp(B)			
Variable	Estimate	*SE*	Lower	Upper	Exp(B)	Lower	Upper	z	*p*
Intercept	0.53401	0.0355	0.4644	0.6036	1.706	1.591	1.83	15.0419	<.001
Gender	−0.01304	0.0656	−0.1416	0.1156	0.987	0.868	1.12	−0.1988	.842
Part of speech mismatch	−0.05371	0.0654	−0.1818	0.0744	0.948	0.834	1.08	−0.8217	.411
Inverse trial	0.58579	0.0742	0.4405	0.7311	1.796	1.553	2.08	7.8998	<.001
Age	0.04093	0.0327	−0.0232	0.1051	1.042	0.977	1.11	1.2503	.211
Cognate	0.17632	0.0406	0.0967	0.2559	1.193	1.102	1.29	4.3402	<.001
Semantic distance	0.01863	0.0313	−0.0428	0.08	1.019	0.958	1.08	0.5946	.552
Edit phon. similarity	0.19763	0.0493	0.101	0.2942	1.219	1.106	1.34	4.0099	<.001
Length agreement	0.30526	0.0504	0.2066	0.404	1.357	1.229	1.5	6.0617	<.001
Frequency (log) agreement	0.1128	0.0345	0.0452	0.1804	1.119	1.046	1.2	3.2686	.001
Trial order	−0.00206	0.0315	−0.0639	0.0598	0.998	0.938	1.06	−0.0652	.948
Vector phon. similarity	0.10841	0.0353	0.0393	0.1776	1.115	1.04	1.19	3.0731	.002

**Table B6.** Detailed results—Latvian.

			95% Conf. Int.			95% Conf. Int.			
			Estimate			Exp(B)			
Variable	Estimate	*SE*	Lower	Upper	Exp(B)	Lower	Upper	z	*p*
Intercept	1.9867	0.08	1.8298	2.1436	7.291	6.233	8.53	24.822	<.001
Gender	−0.0322	0.1	−0.2282	0.1638	0.968	0.796	1.18	−0.322	.747
Part of speech mismatch	0.0368	0.0927	−0.1448	0.2184	1.037	0.865	1.24	0.397	.691
Inverse trial	0.5213	0.0927	0.3396	0.7029	1.684	1.404	2.02	5.625	<.001
Trial order	0.0614	0.0422	−0.0213	0.1441	1.063	0.979	1.15	1.455	.146
Frequency (log) agreement	0.0778	0.051	−0.0222	0.1778	1.081	0.978	1.19	1.525	.127
Length agreement	0.2891	0.0775	0.1372	0.441	1.335	1.147	1.55	3.73	<.001
Edit phon. similarity	0.4538	0.0843	0.2886	0.619	1.574	1.335	1.86	5.384	<.001
Semantic distance	−0.0141	0.0418	−0.096	0.0677	0.986	0.908	1.07	−0.338	.735
Age	0.065	0.0496	−0.0321	0.1622	1.067	0.968	1.18	1.312	.19
Cognate	0.8013	0.0744	0.6554	0.9472	2.228	1.926	2.58	10.765	<.001
Vector phon. similarity	0.1945	0.0538	0.089	0.2999	1.215	1.093	1.35	3.615	<.001
